# COVID‐19: Understanding the impact of anti‐hypertensive drugs and hydroxychloroquine on the ACE1 and ACE2 in lung and adipose tissue in SHR and WKY rats

**DOI:** 10.14814/phy2.15598

**Published:** 2023-02-07

**Authors:** Beatriz Santos Geoffroy Corrêa, Silvana de Barros, Julia Braga Vaz, Maria Angelica Peres, Mayara Klimuk Uchiyama, Alexandre Alves da Silva, Luzia Naoko Shinohara Furukawa

**Affiliations:** ^1^ Laboratory of Renal Pathophysiology, Department of Internal Medicine, School of Medicine University of São Paulo São Paulo Brazil; ^2^ Hypertension Unit, Renal Division, General Hospital of School of Medicine University of São Paulo São Paulo Brazil; ^3^ Laboratory of Supramolecular Chemistry & Nanotechnology, Department of Fundamental Chemistry, Institute of Chemistry University of São Paulo São Paulo Brazil; ^4^ Department of Physiology and Biophysics University of Mississippi Medical Center Jackson Jackson Mississippi USA

**Keywords:** ACE2, COVID‐19, hydroxychloroquine, lung tissue, spontaneously hypertensive rat

## Abstract

Hypertensive individuals taking anti‐hypertensive drugs from renin‐angiotensin system inhibitors may exhibit a more severe evolution of the disease when contracting the SARS‐CoV‐2 virus (COVID‐19 disease) due to potential increases in ACE2 expression. The study investigated ACE1 and ACE2 axes and hydroxychloroquine in the lungs and adipose tissue of male and female normotensive Wistar Kyoto (WKY) and spontaneously hypertensive rats (SHRs). SHRs were treated with losartan (10 mg/kg/day) or captopril (10 mg/kg/day) for 14 days or 7 days with hydroxychloroquine (200 mg/kg/day) in drinking water. WKY rats were also treated for 7 days with hydroxychloroquine. Blood pressure (BP), protein, and mRNA expression of ACE1 and ACE2 were analyzed in serum, adipose, and lung tissues. Losartan and captopril reduced BP in both sexes in SHR, whereas hydroxychloroquine increased BP in WKY rats. Losartan reduced ACE2 in serum and lungs in both sexes and in adipose tissue of male SHRs. Captopril decreased ACE2 protein in the lung of females and in adipose tissue in both sexes of SHRs. Hydroxychloroquine decreased ACE1 and ACE2 proteins in the lungs in both sexes and adipose tissue in male SHRs. In female WKY rats, ACE2 protein was lower only in the lungs and adipose tissue. Losartan effectively inhibited ACE2 in male and captopril in female SHRs. Hydroxychloroquine inhibited ACE2 in male SHRs and female WKY rats. These results further our understanding of the ACE2 mechanism in patients under renin‐angiotensin anti‐hypertensive therapy and in many trials using hydroxychloroquine for COVID‐19 treatment and potential sex differences in response to drug treatment.

## Graphical abstract

Losartan treatment decreased ACE2 in tissues, mainly in male than female SHR; captopril treatment showed decreased ACE2 in lung tissue of females and both sexes SHR in the adipose tissue. Hydroxychloroquine effectively decreased ACE2 in the lung of both sexes SHR and only in females WKY. And in the adipose tissue, hydroxychloroquine decreased ACE2 in male SHR and female WKY.

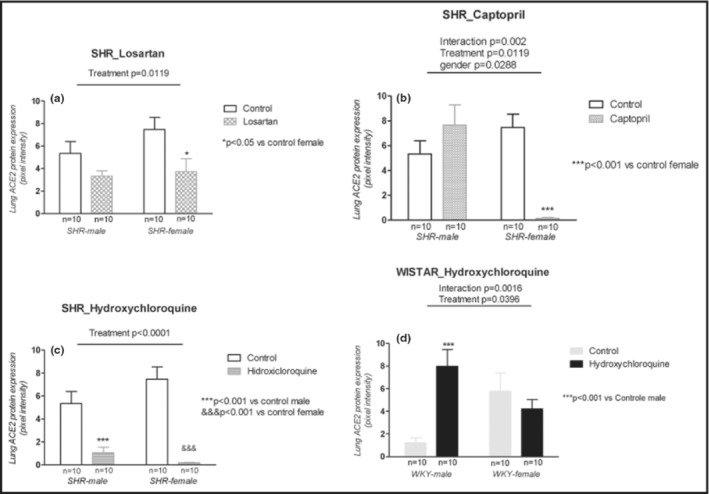

## 1 INTRODUCTION

The COVID‐19 pandemic (coronavirus‐induced disease 2019) of the novel coronavirus (SARS‐CoV‐2) has reached more than 545 million confirmed cases, with a lethality of around 2.0% (WHO, 2010).

SARS‐CoV‐2 depends on its receptor, angiotensin‐converting enzyme 2 (ACE2), to enter cells mediated by its S (spike) glycoprotein for attachment to the host cell membrane and fusion. Once in the host cells, the virus multiplies in the perinuclear area for formation and maturation. Before leaving the infected cell and continuing its multiplying cycle, the S glycoprotein is cleaved by furin in S1 and S2 subunits which remain associated with different functions. In the new target cells, the S1 subunit binds to the receptor, and the S2 subunit anchors the S protein to the virus on the membrane and mediates membrane fusion (Jackson et al., 2022).

Among the various risk factors, advanced age (over 60 years) (Wu et al., 2020; Zhou, Yu, et al., 2020), heart disease, and diabetes mellitus (Yang et al., 2020), hypertension is a concerning factor raised by the scientific community. The anti‐hypertensive treatment with an inhibitor of the renin‐angiotensin system (RAS) axis involves alteration in ACE1 and ACE2 proteins (Bavishi et al., 2016), thus, raising the question of whether increased ACE2 availability could augment the risk for COVID‐19 infection (Ingraham et al., 2020; Watkins, 2020).

According to studies in mice, ACE2 is expressed in all tissues (Gembardt et al., 2005; Hamming et al., 2004), and in the lung, it is abundantly expressed on the apical surface of ciliated epithelial cells that depending on the degree of cell differentiation, may facilitate the entry of the virus into the cells (Jia et al., 2005). Liu et al. (2020) demonstrated increased circulating Ang II in COVID‐19 patients, which could result from decreased ACE2 due to its binding to the SARS‐CoV‐2 S protein.

Anti‐hypertensive drugs that renin and ACE1 lead to a direct decrease in the formation of angiotensin II. At the same time, AT1 inhibition reinforces the negative feedback, inhibiting the entire cascade. A study in normotensive and hypertensive male rats showed that Losartan® (AT1 antagonist) increases ACE2 activity in the heart and kidneys, whereas Captopril® (ACE inhibitor) does not (Ferrario et al., 2005). Studies in lung tissue are scarce, and few studies have evaluated the effects of ACE inhibitors on pulmonary ACE2 in humans or animals.

During this pandemic, clinical evidence recommended the noninterruption of RAS blockers in hypertensive patients. The relationship between these drugs and the increased risk of contracting the virus or worsening disease outcomes is not yet clear (Alexandre et al., 2020; Sanders et al., 2020).

ACE2 is highly expressed in adipose tissue, which can constitute a viral reservoir of SARS‐CoV‐2, exacerbating the severity of COVID‐19 through the amplification of immune cells and cytokine activation (Ryan & Caplice, 2020). Obese patients have more adipose tissue than lean individuals, resulting in more ACE2 receptors (Sabri et al., 1674‐1692). However, the association between obesity and viral load has not yet been confirmed (Argyropoulos et al., 2020).

Chloroquine (CQ) and Hydroxychloroquine (HCQ) block viral entry into cells by inhibiting host receptor glycosylation and proteolytic processes. These drugs also have immunomodulatory effects by attenuating cytokine production and inhibiting autophagy and lysosomal activity in host cells (Devaux et al., 2020; Zhou, Dai, & Tong, 2020). Multiple clinical trials are proposed to investigate the effect of HCQ on COVID‐19 (Yao et al., 2020) because they are low‐cost and promptly available worldwide (Gbinigie & Frie, 2020; Sinha & Balayla, 2020; Yazdany & Kim, 2020). Studies in China concluded that CQ reduced lung pathology and shortened the disease course without severe adverse reactions (Chen et al., 2020; Gao et al., 2020); studies in France suggested that HCQ, especially when combined with azithromycin, can reduce the viral load in COVID‐19 patients (Gautret, Lagier, Parola, Hoang, Meddeb, Mailhe, Doudier, Aubry, et al., 2020; Gautret, Lagier, Parola, Hoang, Meddeb, Mailhe, Doudier, Courjon, et al., 2020). Furthermore, the Indian Council of Medical Research recommended HCQ for chemoprophylaxis of asymptomatic health workers and households in India (Nina & Dash, 2020). However, other clinical studies showed no reduction in hospitalized patients (RECOVERY Collaborative Group et al., 2020) receiving HCQ treatment. A recent systematic study with meta‐analysis demonstrated that HCQ should not be ruled out for COVID‐19 treatment because results from clinical trials are still inconclusive for HCQ effectiveness (García‐Albéniz et al., 2022).

The main goal of the current study was to investigate ACE and ACE2 axes in lungs and adipose tissue during RAS inhibition and HCQ treatment in normotensive and hypertensive rats. Male and female rats were also studied to examine the impact of gender on ACE and ACE2 axes in response to these treatments (Gebhard et al., 2020; Mourosi et al., 2022).

## 2 MATERIALS AND METHODS

### 2.1 Animals

Juvenile 6‐week‐old male and female normotensive Wistar Kyoto (WKY) and spontaneously hypertensive rats (SHRs) were purchased from the ICB/USP Animal Care Center. The experimental protocol was approved by the CEUA (Ethics Committee on the Use of Animals from the University of São Paulo School of Medicine no. 1535/2020). During the execution of the experimental protocols, rats were kept in a controlled temperature environment with a 12/12‐hour day/night cycle.

### 2.2 Experimental protocol

At 9 weeks of age, male and female SHRs were divided into four experimental groups (*n* = 10 males and 10 females per group) to receive the following treatments: (1) control: filtered water; (2) angiotensin‐converting enzyme inhibitor (Captopril) 10 mg/kg/day in drinking water for 14 days; (3) angiotensin I receptor blocker (ARB), losartan, 10 mg/Kg/day, in drinking water for 14 days; or (4) Hydroxychloroquine (HCQ) treatment which started at 10 weeks of age at 100 mg/kg in drinking water for 7 days.

At 10 weeks of age, male and female WKY rats were divided into two experimental groups (*n* = 10 males and 10 females per group) to receive either vehicle control (filtered water) or Hydroxychloroquine (HCQ; 100 mg/kg in drinking water for 7 days).

### 2.3 Tail‐cuff blood pressure measurement

The tail‐cuff blood pressure (BP) was measured before and after treatment by a noninvasive blood pressure analyzer that uses light transmission photoplethysmography to determine BP and heart rate (BP‐2000 Series II Blood Pressure Analysis System, Visitech System, Inc.).

### 2.4 Serum levels of ACE1 and ACE2

Serum was obtained from blood samples collected at the end of treatment from SHR and WKY rats by tail vein puncture to determine ACE1 and ACE2 concentration using Rat ACE (Angiotensin I Converting Enzyme) ELISA Kit and Rat ACE2 (Angiotensin‐Converting Enzyme 2) ELISA Kit from Elabscience.

### 2.5 Tissue ACE1 and ACE2 immunohistochemistry

Adipose and lung tissue samples were collected after euthanasia (150 mg/kg intraperitoneal, sodium pentobarbital, Thiopentax, Cristalia) at the end of treatment. Tissue samples were fixed in 10% formalin‐phosphate buffer and processed for paraffin inclusion. We obtained sections of 4.5 μm from each tissue using a microtome. The tissue sections were sealed in glass slides and submitted to immunohistochemistry using an anti‐ACE Antibody (2E2: sc‐23908) and anti‐ACE2 antibody (E‐11: sc‐390851) from Santa Cruz and revealed using Dako EnVision® + Dual Link System‐HRP (DAB+). The semi‐quantitative protein expression was evaluated by ImageJ Fiji software (https://imagej.net/Fiji/Downloads).

### 2.6 Tissue mRNA ACE1 and mRNA ACE2 expression

Relative mRNA ACE1 and ACE2 expression were analyzed after total RNA was extracted from adipose and lung tissues using the TRizol method (Thermo Fisher Scientific). Total RNA was transcripted to cDNA by reverse transcriptase method (Improm II, Promega) to quantify the mRNA ACE (NCBI Reference Sequence: NM_012544.1) and ACE2 (NCBI Reference Sequence: NM_001012006.2) using correspondent primers by qPCR method (QuantiNova SYBR Green PCR Kit, QIAGEN). The primers were designed by the Integrated DNA Technologies IDT program (www.idtdna.com) and synthesized by Extend (www.exxtend.com.br). The reference gene chosen was β‐actin (NCBI Reference Sequence: NM_031144.3). The quantification for gene expression was performed by the ΔΔCT method of relative quantification.

### 2.7 Statistical analysis

Statistical analyses were performed using the GraphPad Prism 5 program. Two‐way ANOVA with no repeated measures with two factors (treatment and gender) followed by an ad‐hoc test was used to identify differences between the analyzed groups. The data of each variable were expressed as mean ± standard error. The number of animals was calculated based on previous studies from our group and preliminary experiments suggesting that an *n* = 10 per group was sufficient to achieve a power (1‐beta) of 0.80–0.85 and a probability of a type I error (alpha) of 0.05. Values of *p* < 0.05 were considered significant.

## 3 RESULTS

### 3.1 Losartan treatment in SHR

Losartan treatment in SHR reduced systolic blood pressure (*p* < 0.0001) and adipose tissue mass (*p* = 0.0225) in both sexes (Table 1).

**TABLE 1:** Characteristics of male and female SHR treated with losartan.

Parameter	Male—SHR		Female—SHR		Two‐way (*p* value)		
	Control (*n* = 10)	Losartan (*n* = 10)	Control (*n* = 10)	Losartan (*n* = 10)	Interaction	Treatment	Gender
Body weight (g)	242.3 ± 4.8	238.3 ± 3.1	153.5 ± 3.3	159.7 ± 2.6	0.1594	0.7526	<0.0001
Systolic blood pressure (mm Hg)	205.2 ± 6.1	152.3 ± 3.3	182.8 ± 3.1	142.7 ± 2.9	0.1248	<0.0001	0.0004
Adipose tissue mass (g/100 g body weight)	0.89 ± 0.030	0.78 ± 0.025	0.43 ± 0.039	0.40 ± 0.027	0.1937	0.0225	<0.0001

*Note*: SHR—spontaneously hypertensive rat; values are means ± SE; two‐way ANOVA, *p* < 0.05.

### 3.2 Captopril treatment in SHR

As shown in Table 2, similar to what was observed with losartan, captopril also reduced BP (*p* < 0.0001) and adiposity (*p* < 0.0001) in both sexes. Captopril treatment evoked distinct effects on body weight in males (reduction) versus females (increase) SHR (interaction, *p* = 0.0148).

**TABLE 2:** Characteristics of male and female SHR treated with captopril.

Parameter	Male—SHR		Female—SHR		Two‐way (*p* value)		
	Control (*n* = 10)	Captopril (*n* = 10)	Control (*n* = 10)	Captopril (*n* = 10)	Interaction	Treatment	Gender
Body weight (g)	242.3 ± 4.8	234.1 ± 3.3	153.5 ± 3.3	163.5 ± 2.3	0.0148	0.7937	<0.0001
Systolic pressure (mm Hg)	205.2 ± 6.1	150.0 ± 3.0	182.8 ± 3.1	142.2 ± 5.7	0.1306	<0.0001	0.0028
Adipose tissue mass (g/100 g body weight)	0.89 ± 0.030	0.64 ± 0.033	0.43 ± 0.039	0.38 ± 0.020	0.0023	<0.0001	<0.0001

*Note*: SHR—spontaneously hypertensive rat; values are means ± SE; two‐way ANOVA, *p* < 0.05.

### 3.3 Hydroxychloroquine (HCQ) treatment in SHR

Hydroxychloroquine treatment in SHRs showed significant interaction (*p* < 0.0001) with reduced body weight in males but not females. Moreover, it reduced adipose tissue mass (*p* < 0.0001) in both sexes (Table 3). BP was not different from the control groups in both sexes.

**TABLE 3:** Characteristics of male and female SHR treated with Hydroxychloroquine.

Parameter	Male—SHR		Female—SHR		Two‐way (p value)		
	Control (*n* = 10)	HCQ (*n* = 10)	Control (*n* = 10)	HCQ (*n* = 10)	Interaction	Treatment	Gender
Body weight (g)	242.3 ± 4.8	192.2 ± 7.3	153.5 ± 3.3	149.9 ± 1.5	<0.0001	<0.0001	<0.0001
Systolic blood pressure (mm Hg)	205.2 ± 6.1	193.3 ± 4.1	182.8 ± 3.1	181.8 ± 5.8	0.2781	0.2001	0.0016
Adipose tissue mass (g/100 g body weight)	0.89 ± 0.030	0.32 ± 0.038	0.43 ± 0.039	0.25 ± 0.010	<0.0001	<0.0001	<0.0001

*Note*: SHR—spontaneously hypertensive rat; values are means ± SE; two‐way ANOVA, *p* < 0.05.

### 3.4 Hydroxychloroquine (HCQ) treatment in WKY rats

The hydroxychloroquine treatment in WKY rats reduced body weight (*p* < 0.0001), and in contrast, the systolic BP increased in both sexes (*p* = 0.0061). Also, the adipose tissue mass (*p* < 0.0001) decreased in both sexes (Table 4).

**TABLE 4:** Characteristics of male and female WKY treated with Hydroxychloroquine.

Parameter	Male—WKY		Female—WKY		Two‐way (p value)		
	Control (*n* = 10)	HCQ (*n* = 10)	Control (*n* = 10)	HCQ (*n* = 10)	Interaction	Treatment	Gender
Body weight (g)	264.5 ± 4.7	238.9 ± 4.80	190.0 ± 2.1	164.3 ± 2.1	0.9985	<0.0001	<0.0001
Systolic blood pressure (mm Hg)	153.6 ± 3.5	166.0 ± 4.0	148.5 ± 4.4	157.8 ± 2.7	0.6797	0.0061	0.0824
Adipose tissue mass (g/100 g body weight)	0.85 ± 0.028	0.60 ± 0.040	0.81 ± 0.033	0.61 ± 0.031	0.4269	<0.0001	0.6422

*Note*: WKY—normotensive Wistar Kyoto rat; values are means ± SE; two‐way ANOVA, *p* < 0.05.

### 3.5 Serum ACE1

**FIGURE 1:**
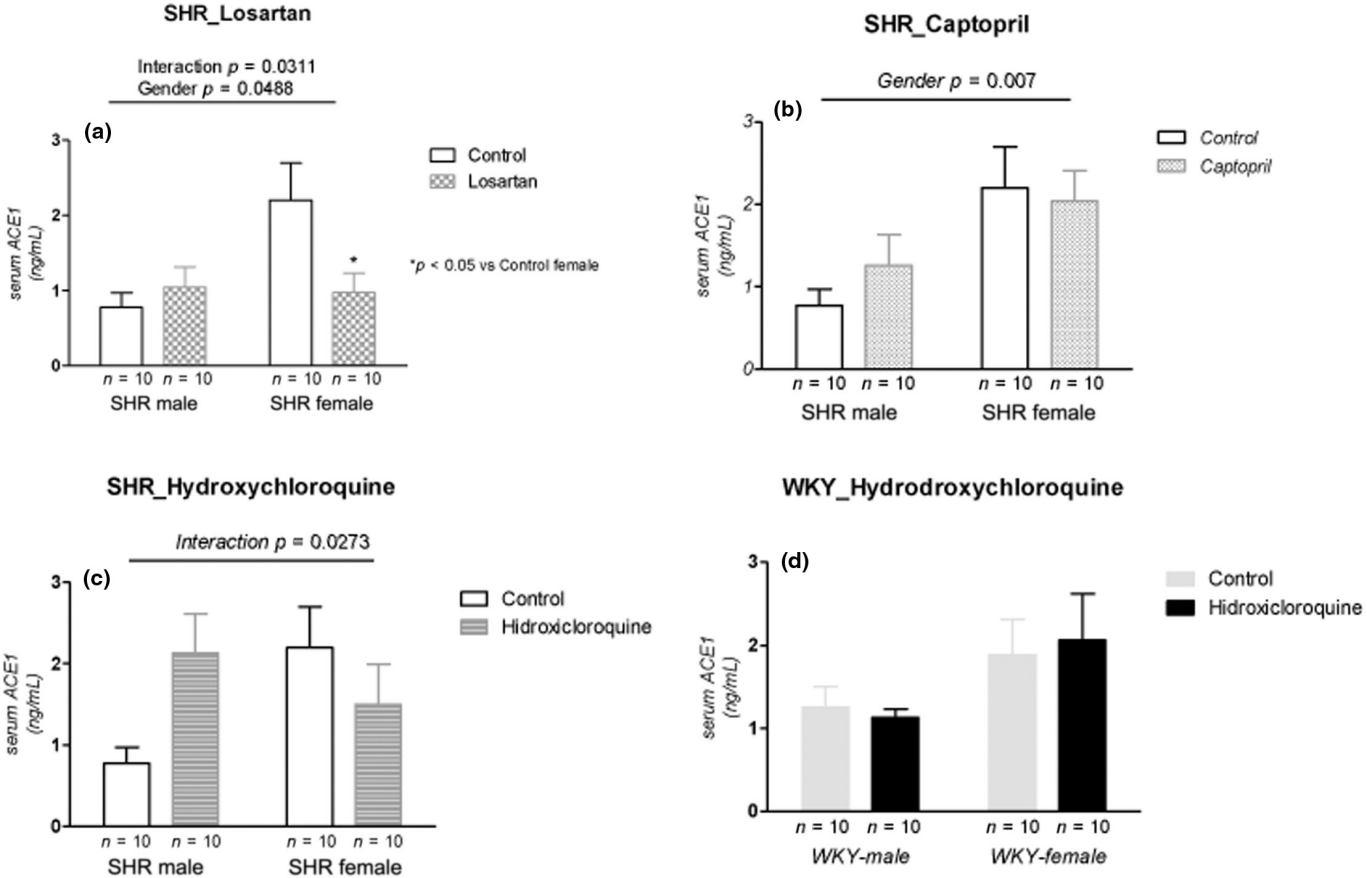
Serum ACE1 (ng/mL) in male and female SHR rats treated with Losartan (a), Captopril (b), and Hydroxychloroquine (c) and in male and female WKY rats treated with Hydroxychloroquine (d). Data are presented as mean ± SE and analyzed with two‐way ANOVA, with no repeated measures and a significance of *p* < 0.05. Each experimental group of males and females is composed of *n* = 10 animals.

Losartan treatment showed significant interaction with increased serum ACE1 in the male and decreased it in the female SHR (*p* = 0.0311; Figure 1a), whereas the captopril treatment showed a gender difference (*p* = 0.007), in which serum ACE1 was higher in females than males (Figure 1b). Similar to losartan, Hydroxychloroquine treatment showed interaction with increased serum ACE1 in males and reduced it in females SHR (*p* = 0.0273; Figure 1c). In normotensive WKY rats, serum ACE1 was not different with Hydroxychloroquine treatment in both sexes (Figure 1d).

### 3.6 Serum ACE2

**FIGURE 2:**
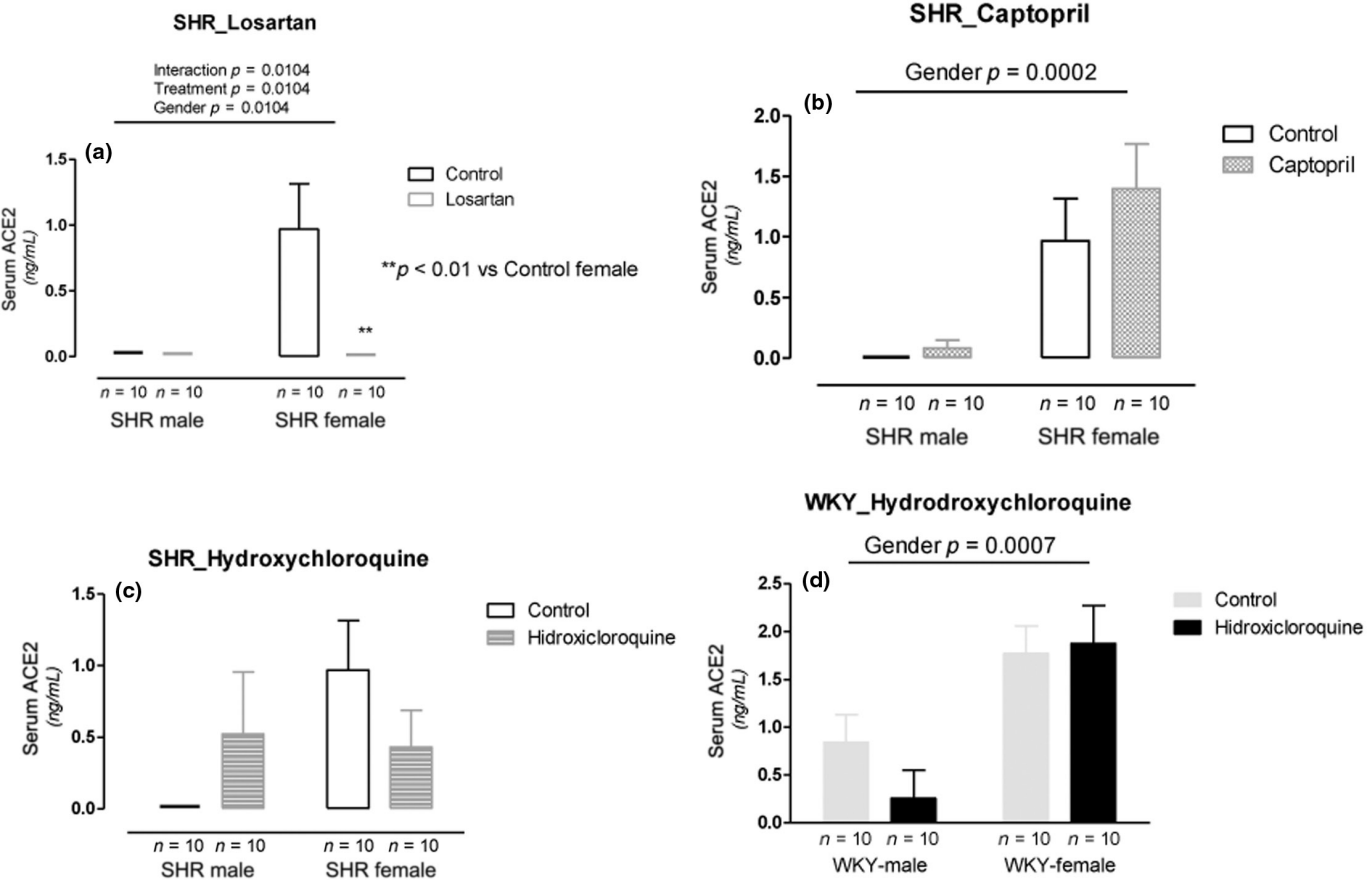
Serum ACE2 (ng/mL) in male and female SHR rats treated with Losartan (a), Captopril (b), and Hydroxychloroquine (c) and in male and female WKY rats treated with Hydroxychloroquine (d). Data are presented as mean ± SE and analyzed with two‐way ANOVA, with no repeated measures and a significance of *p* < 0.05. Each experimental group of males and females is composed of *n* = 10 animals.

Losartan treatment showed interaction with no change in serum ACE2 in the males and decreased in the females (*p* = 0.0104). Interestingly, serum ACE2 was practically undetectable at baseline in the males and became undetectable with losartan treatment in the female SHR (Figure 2a). In the captopril treatment, we observed gender differences (*p* = 0.0002) in which serum ACE2 was higher in females than in males SHR (Figure 2b). Hydroxychloroquine treatment did not change serum ACE2 in both sexes of SHRs (Figure 2c). In normotensive WKY rats, we observed gender differences (*p* = 0.0007), where serum ACE2 was higher in females than in males (Figure 2d).

### 3.7 Lung—ACE1 protein expression

**FIGURE 3:**
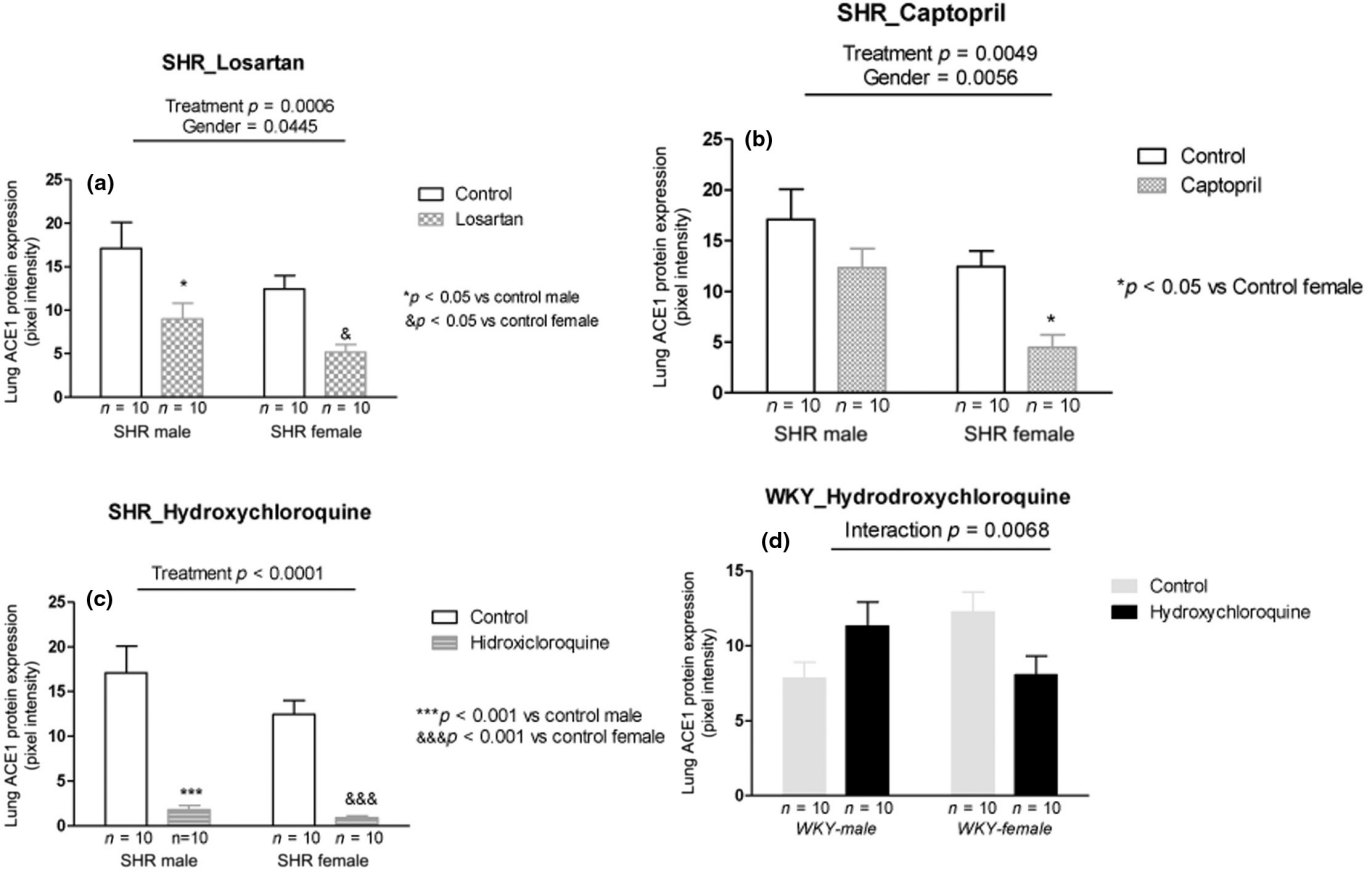
ACE1 protein expression (pixel intensity) in the lung tissue of male and female SHR rats treated with Losartan (a), Captopril (b), and Hydroxychloroquine (c) and in male and female WKY rats treated with Hydroxychloroquine (d). Data are presented as mean ± SE and analyzed with two‐way ANOVA, with no repeated measures and a significance of *p* < 0.05. Each experimental group of males and females is composed of *n* = 10 animals.

**FIGURE 4:**
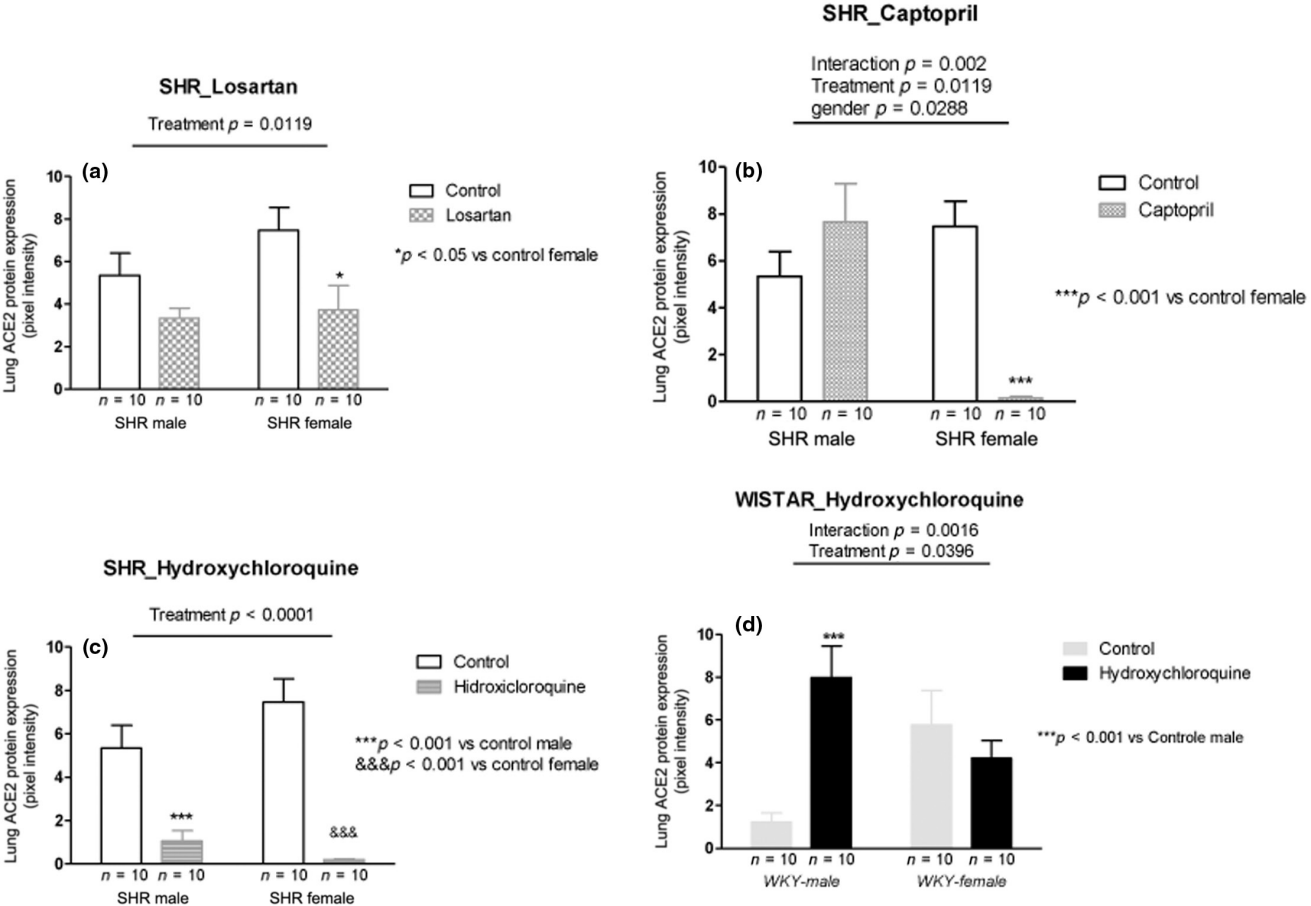
ACE2 protein expression (pixel intensity) in the lung tissue of male and female SHR rats treated with Losartan (a), Captopril (b), and Hydroxychloroquine (c) and in male and female WKY rats treated with Hydroxychloroquine (d). Data are presented as mean ± SE and analyzed with two‐way ANOVA, with no repeated measures and a significance of *p* < 0.05. Each experimental group of males and females is composed of *n* = 10 animals.

Losartan treatment reduced protein ACE1 expression in male and female SHRs compared with their respective controls (*p* = 0.0006; Figure 3a). Similar to losartan treatment, captopril reduced ACE1 protein expression in both males and females (*p* = 0.0049; Figure 3b). Also, HCQ treatment reduced protein ACE1 expression significantly in males and females SHR (*p* < 0.0001; Figure 3c). In the normotensive WKY rats, the hydroxychloroquine treatment showed interaction with increased protein ACE1 expression in the males and reduced in the females (*p* = 0.0068; Figure 4d).

### 3.8 Lung—ACE2 protein expression

Losartan treatment decreased ACE2 protein expression in male and female SHRs (*p* = 0.0119; Figure 4a). Captopril treatment showed significant interaction with increased ACE2 protein expression in males and decreased in female SHRs (*p* = 0.002; Figure 4b). Similar to losartan, HCQ treatment significantly reduced ACE2 protein expression in male and female SHRs (*p* < 0.0001; Figure 4c). Different from SHRs, in normotensive WKY rats, HCQ treatment showed significant interaction with increased ACE2 protein expression in males and decreased it in female WKY rats (*p* = 0.0016; Figure 4d).

### 3.9 Lung—ACE1 mRNA expression

**FIGURE 5:**
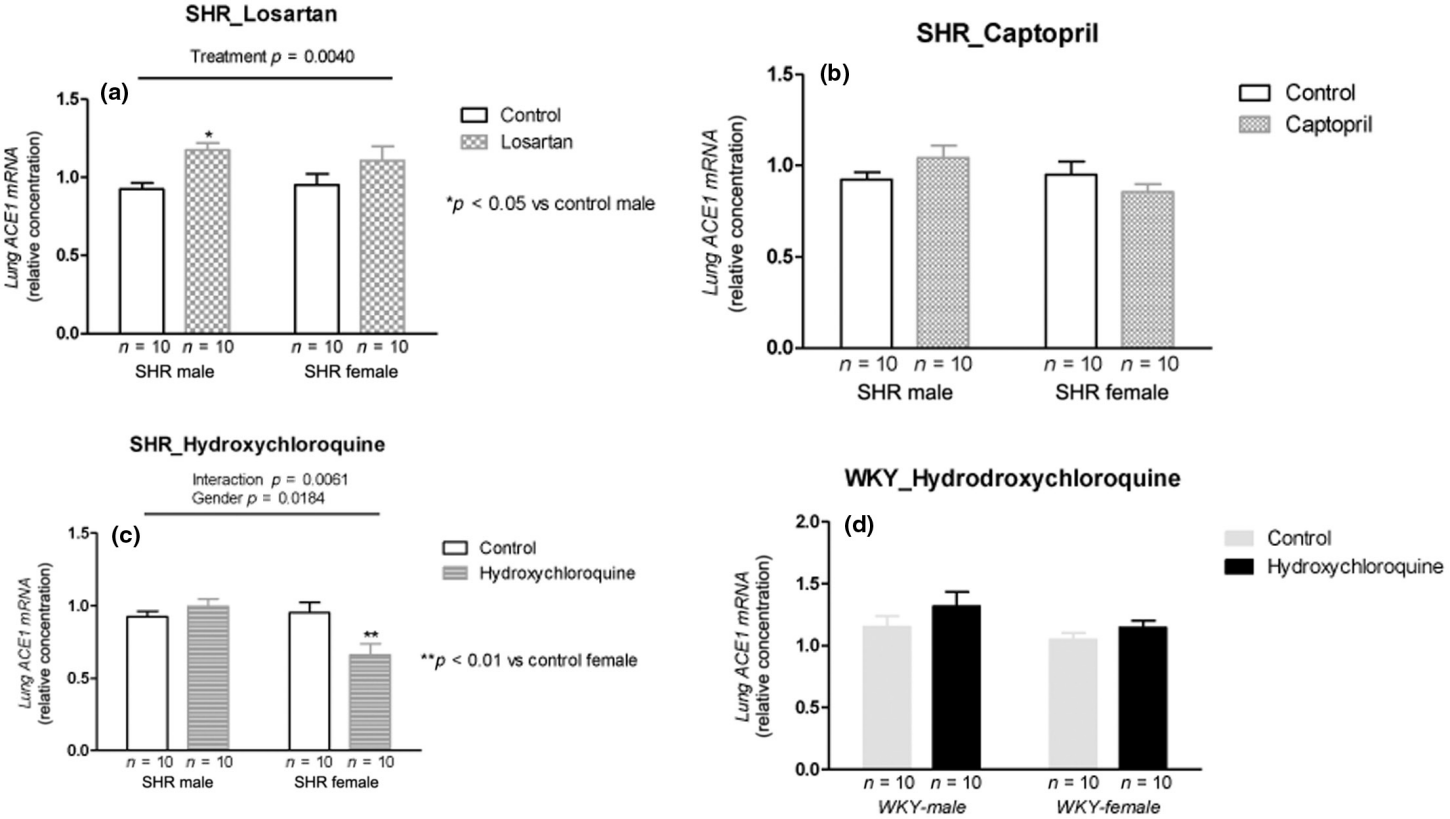
ACE1 mRNA expression (relative concentration) in the lung tissue of male and female SHR rats treated with Losartan (a), Captopril (b), and Hydroxychloroquine (c) and in male and female WKY rats treated with Hydroxychloroquine (d). Data are presented as mean ± SE and analyzed with two‐way ANOVA, with no repeated measures and a significance of *p* < 0.05. Each experimental group of males and females is composed of *n* = 10 animals.

Losartan treatment increased ACE1 mRNA expression in male and female SHRs (*p* = 0.0040; Figure 5a). Captopril treatment did not change ACE1 mRNA expression in SHRs (Figure 5b). HCQ treatment showed interaction with increased mRNA ACE1 expression in males and decreased in the female SHRs (*p* = 0.0061; Figure 5c). In WKY rats, HCQ treatment did not change ACE1 mRNA expression in both sexes (Figure 5d).

### 3.10 Lung—ACE2 mRNA expression

**FIGURE 6:**
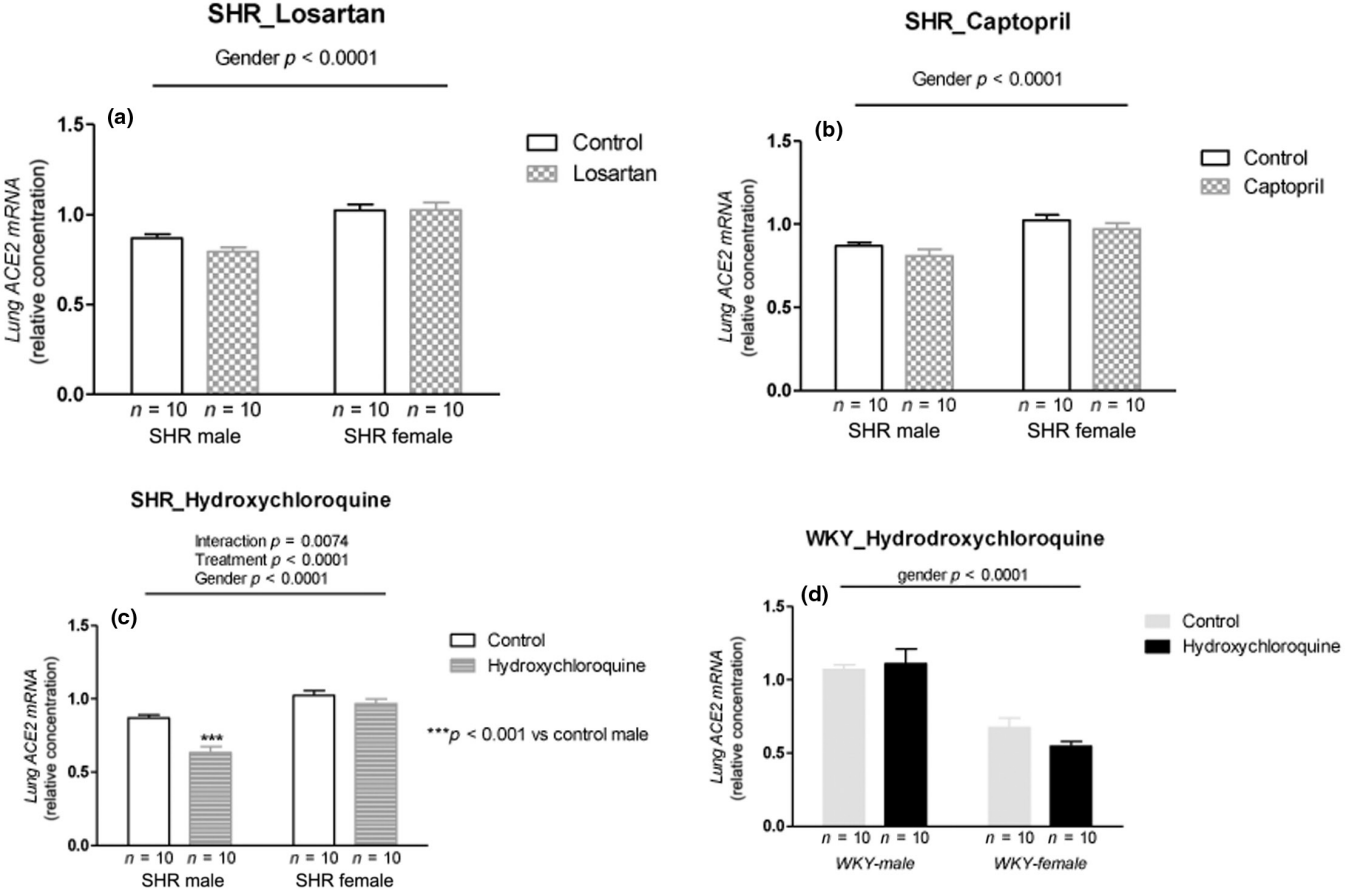
ACE2 mRNA expression (relative concentration) in the lung tissue of male and female SHR rats treated with Losartan (a), Captopril (b), and Hydroxychloroquine (c) and in male and female WKY rats treated with Hydroxychloroquine (d). Data are presented as mean ± SE and analyzed with two‐way ANOVA, with no repeated measures and a significance of *p* < 0.05. Each experimental group of males and females is composed of *n* = 10 animals.

Losartan and captopril treatment did not change ACE2 mRNA expression in SHRs; however, we observed gender differences in which females showed higher levels than males (*p* < 0.0001; Figure 6a and *p* < 0.0001; Figure 6b). HCQ treatment showed interaction with decreased ACE2 mRNA expression in males but caused no change in female SHRs (*p* = 0.0061; Figure 6c). In WKY rats, HCQ treatment did not change ACE2 mRNA expression in both sexes, although we observed higher values in the males compared with female WKY rats (*p* < 0.0001; Figure 6d).

### 3.11 Adipose tissue—ACE1 protein expression

**FIGURE 7:**
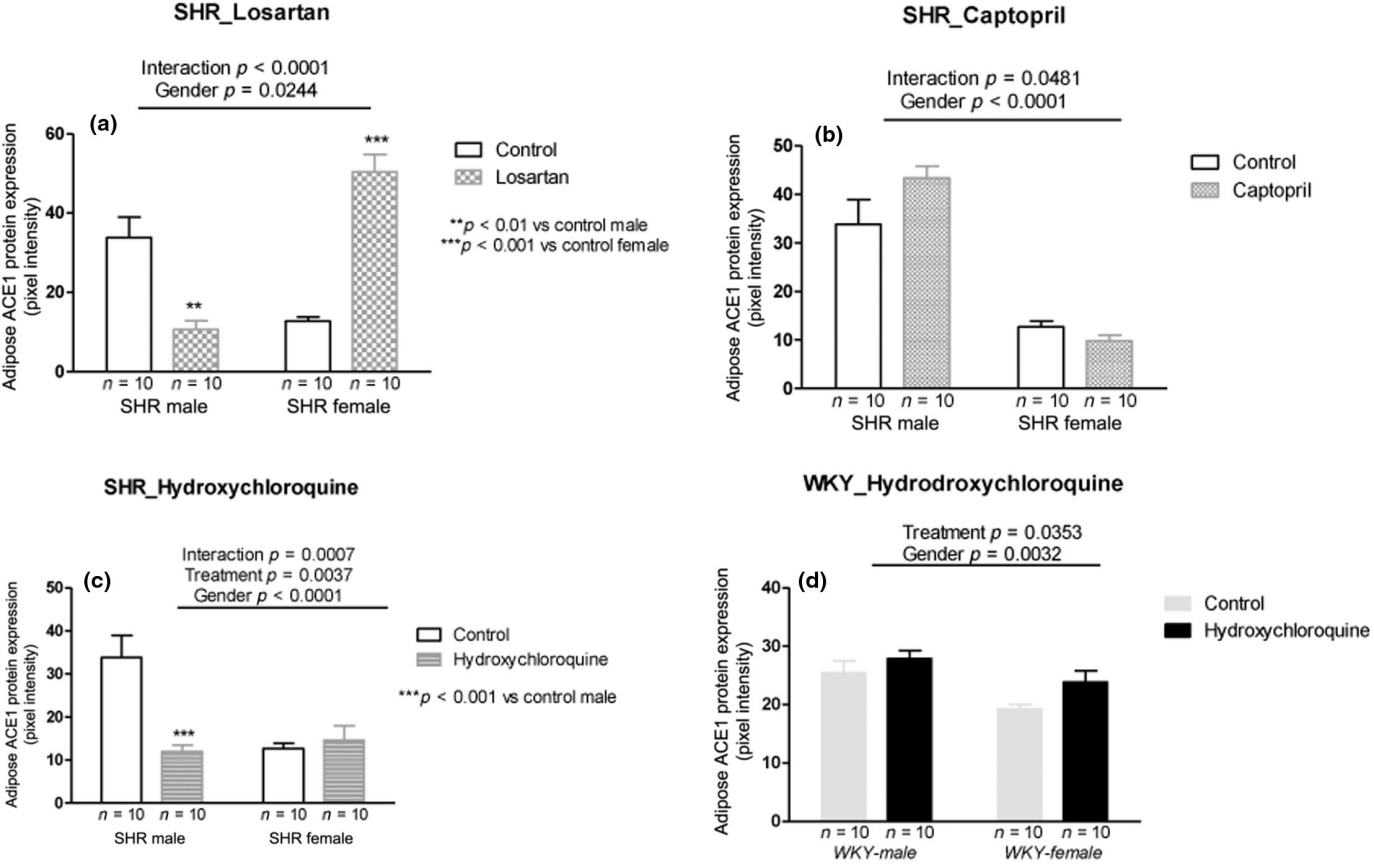
ACE1 protein expression (pixel intensity) in the adipose tissue of male and female SHR rats treated with Losartan (a), Captopril (b), and Hydroxychloroquine (c) and in male and female WKY rats treated with Hydroxychloroquine (d). Data are presented as mean ± SE and analyzed with two‐way ANOVA, with no repeated measures and a significance of *p* < 0.05. Each experimental group of males and females is composed of *n* = 10 animals.

Losartan treatment showed interaction with decreased ACE1 protein expression in males and increased it in female SHRs (*p* < 0.001; Figure 7a). Captopril treatment also increased ACE1 protein expression in males but did not change in female SHRs (interaction *p* = 0.0481; Figure 7b). HCQ treatment showed interaction with decreased ACE1 protein expression in males and no change in female SHRs (*p* = 0.0007; Figure 7c). In normotensive WKY rats, HCQ treatment increased the ACE1 protein expression in both sexes (*p* = 0.0353; Figure 7d).

### 3.12 Adipose tissue—ACE2 protein expression

**FIGURE 8:**
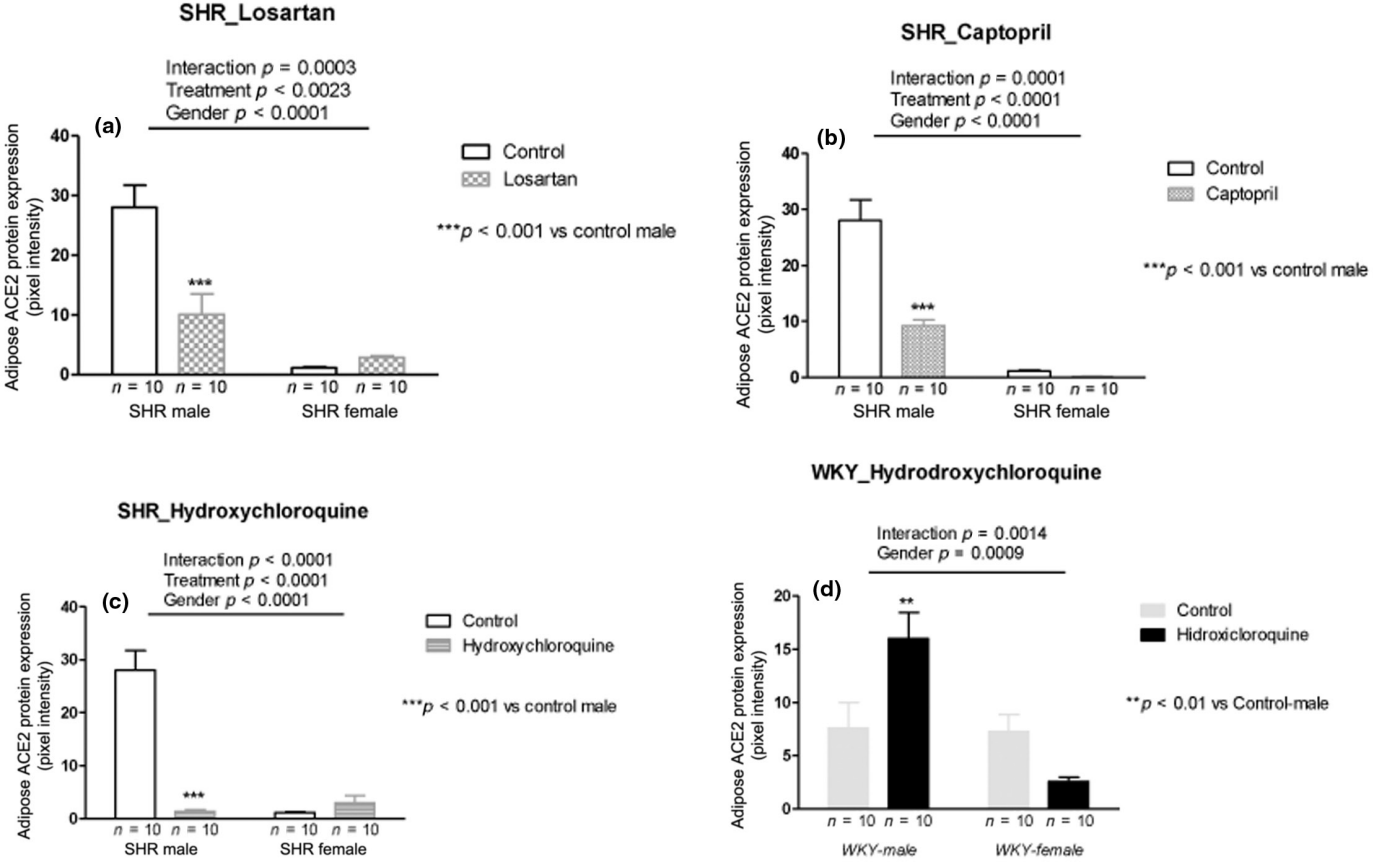
ACE2 protein expression (pixel intensity) in the adipose tissue of male and female SHR rats treated with Losartan (a), Captopril (b), and Hydroxychloroquine (c) and in male and female WKY rats treated with Hydroxychloroquine (d). Data are presented as mean ± SE and analyzed with two‐way ANOVA, with no repeated measures and a significance of *p* < 0.05. Each experimental group of males and females is composed of *n* = 10 animals.

Losartan treatment showed interaction with decreased ACE2 protein expression in males and increased in female SHRs (*p* = 0.0003; Figure 8a). Captopril treatment showed interaction with decreased ACE2 protein expression in males and no difference in female SHRs (*p* = 0.0001; Figure 8b). HCQ treatment showed interaction with decreased ACE2 protein expression in the males and increased in the female SHRs (*p* < 0.0001; Figure 8c). In normotensive WKY rats, HCQ treatment showed interaction with increased ACE2 protein expression in the males and decreased in females (*p* = 0.0014; Figure 8d).

### 3.13 Adipose tissue—ACE1 mRNA expression

**FIGURE 9:**
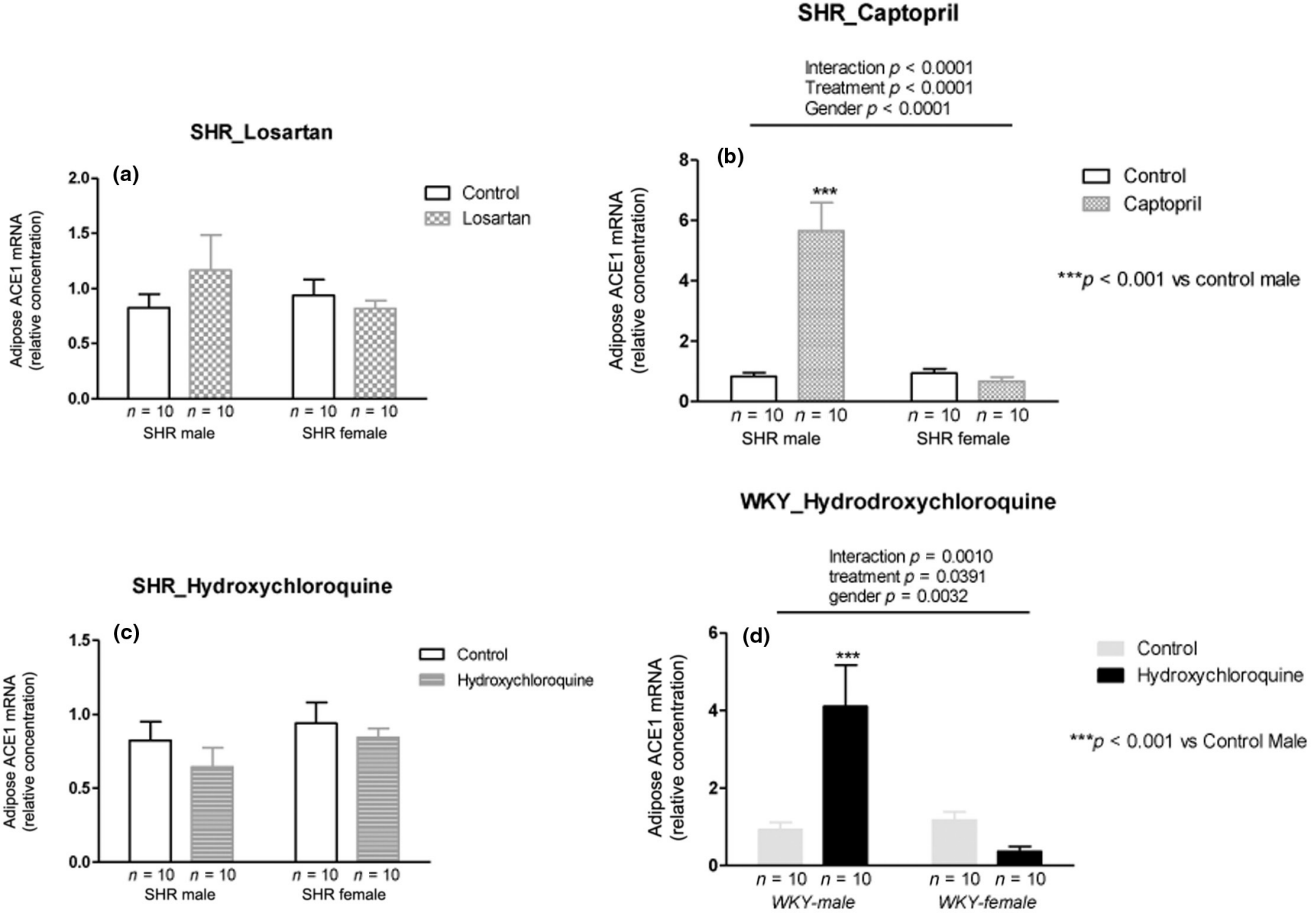
ACE1 mRNA expression (relative concentration) in the adipose tissue of male and female SHR rats treated with Losartan (a), Captopril (b), and Hydroxychloroquine (c) and in male and female WKY rats treated with Hydroxychloroquine (d). Data are presented as mean ± SE and analyzed with two‐way ANOVA, with no repeated measures and a significance of *p* < 0.05. Each experimental group of males and females is composed of *n* = 10 animals.

Losartan treatment did not change the ACE1 mRNA expression in both sexes in SHRs (Figure 9a). Captopril treatment showed interaction with an increase in the males (*p* < 0.001) and a decrease in female SHRs (*p* < 0.0001; Figure 9b). HCQ treatment did not change ACE1 mRNA expression in SHRs (Figure 9c). In normotensive WKY rats, HCQ treatment showed interaction with increased ACE1 mRNA expression in the males and decreased in females (*p* = 0.001; Figure 9d).

### 3.14 Adipose tissue—ACE2 mRNA expression

**FIGURE 10:**
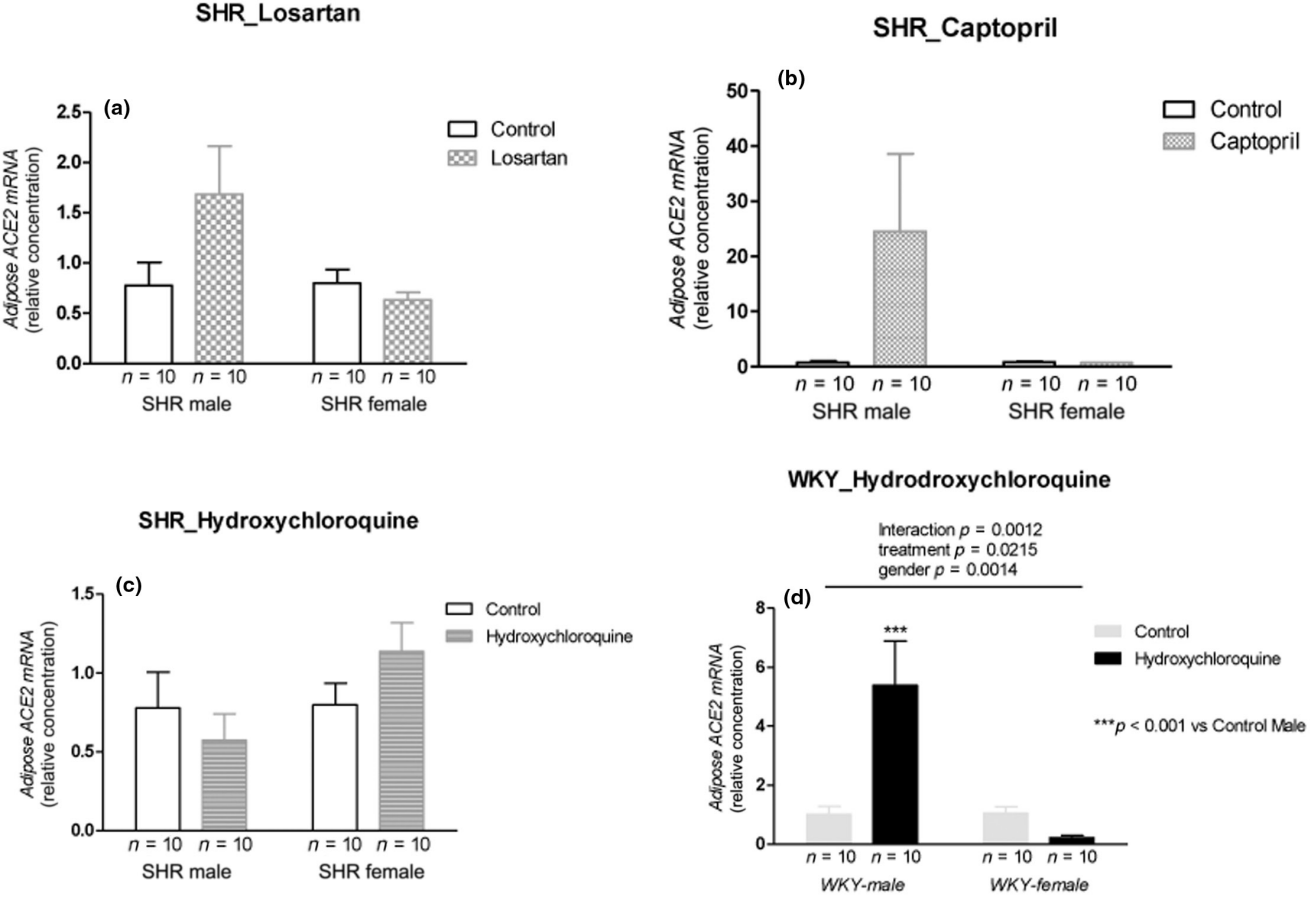
ACE2 mRNA expression (relative concentration) in the adipose tissue of male and female SHR rats treated with Losartan (a), Captopril (b), and Hydroxychloroquine (c) and in male and female WKY rats treated with Hydroxychloroquine (d). Data are presented as mean ± SE and analyzed with two‐way ANOVA, with no repeated measures and a significance of *p* < 0.05. Each experimental group of males and females is composed of *n* = 10 animals.

The treatment with losartan, captopril, and HCQ did not change ACE2 mRNA expression in adipose tissue in both sexes in SHRs (Figure 10a–c). In normotensive WKY rats, HCQ showed interaction with increased ACE2 mRNA expression in the males and decreased in females (*p* = 0.0012; Figure 10d).

## 4 DISCUSSION

Our main goal was to investigate the modulation of ACE2 by losartan (AT1 inhibitor), and captopril (ACE1 inhibitor), renin‐angiotensin anti‐hypertensive drugs in males and females is based on the clinical evidence that renin‐angiotensin anti‐hypertensive drugs could increase the availability of ACE2 which could, in turn, increase the risk of COVID‐19 infection since SARS‐CoV‐2 virus uses ACE2 as a receptor to enter the cells and to trigger its infectious cycle (Ingraham et al., 2020; Watkins, 2020).

In addition to the effective decrease in BP with losartan (Kristek et al., 2013; Nunez et al., 1997), we observed a reduction in adipose tissue mass without body weight change in both sexes in SHRs.

We observed an effective reduction of serum ACE2 in both sexes in SHRs with losartan treatment. In lung tissue, losartan treatment reduced ACE1 and ACE2 protein expression in both sexes in SHRs. The increase in ACE1 mRNA expression in both sexes was probably due to a counter‐regulatory mechanism in response to the inhibition of ACE1 protein; however, ACE2 mRNA expression did not change in both sexes with losartan treatment. Otherwise, losartan treatment acts differently in adipose tissue compared with lung tissue, where ACE1 and ACE2 protein expression decreased in males and increased in female SHRs. ACE1 and ACE2 mRNA expression in the adipose tissue was not changed with losartan treatment in both sexes of SHRs. These results show that losartan decreases ACE2 in serum and lungs of both sexes, while in the adipose tissue only in males, with increases in female SHRs. Another exciting result is that losartan treatment changes the ACE1 mRNA in both tissues, while ACE2 mRNA did not change in these tissues. Taking into account, the mechanism of the SARS‐CoV‐2 virus entering the cell via the ACE2 receptor in the cell membrane (Jackson et al., 2022), the present data suggest that losartan effectively reduces ACE2 in blood, lungs, and adipose tissue. Ferrario et al. (Ferrario et al., 2005) observed that the ACE2 mRNA increased in the heart during ACE inhibition or losartan treatment, which corroborates the results from Sommerstein and Grani (Sommerstein & Gräni, 2020) that ACE inhibitors may be a potential risk factor for fatal COVID‐19. In the present study, we demonstrated that losartan could be considered the drug of choice that protects against SARS‐CoV‐2 infection, at least in hypertensive patients and mainly in males compared with females.

Captopril treatment effectively reduced BP, similar to losartan treatment. Indeed, captopril treatment‐induced decreases in adipose tissue mass without changing body weight in both sexes of SHR. Captopril treatment reduced ACE1 protein expression in both sexes and protein ACE2 expression in females while increasing it in the lung tissue of male SHRs. Milne and col (Milne et al., 2020) showed that ACE2 mRNA expression is decreased in the human lung under ACEi use; however, in the present study, captopril treatment did not alter ACE1 and ACE2 mRNA expression in lung tissue of both sexes. These data demonstrated that captopril effectively inhibits ACE1 in both sexes. However, it showed sexual dimorphism for ACE2 protein in the lung of SHRs. In the adipose tissue, captopril acts differently from lung tissue, increasing ACE1 protein expression in males and decreasing it in females, while reducing ACE2 protein expression in both sexes in SHRs. ACE1 mRNA expression in the adipose tissue increased in males and decreased in females. Also, ACE2 mRNA increased in males, but was not statistically significant, and no change in female SHRs. Therefore, we observed that captopril acts on ACE1 and ACE2 proteins differently in each tissue and that there is sexual dimorphism. Also, the regulation of ACE1 and ACE2 mRNA expression is different in the tissues and between the sexes. We observed that even though both anti‐hypertensive losartan and captopril act on the renin‐angiotensin system since each one acts in different steps of the system, the mechanism of ECA2 regulation is different.

Also, we evaluated HCQ in male and female normotensive WKY and SHR rats. HCQ drug was chosen in the present study to test whether it was involved with ACE2; this drug was the drug most administered in patients at the beginning of the COVID‐19 pandemic; unfortunately, without any conclusive beneficial effect, it is not included as a cocktail drug for COVID‐19 treatment.

In the present study, it was possible to observe that HCQ increases BP in normotensive WKY rats but not in hypertensive SHRs. Furthermore, it decreases adipose tissue mass in both sexes of WKY and the SHR. Other studies showed that blood pressure decreases with hydroxychloroquine treatment in patients with systemic lupus erythematosus (Reese et al., 2019) and rheumatoid arthritis (Baker et al., 2018), and no change in the control mice group was probably due to the lower concentration of HCQ administration (Gómez‐Guzmán et al., 2014) than in our study.

Surprisingly, HCQ treatment showed effects on the ACE1 and ACE2 in SHR and WKY rats. Serum ACE1 increases in male and decreases in female SHRs, whereas serum ACE2 was not different in both sexes. In the normotensive WKY rat, serum ACE1 and ACE2 did not change with HCQ treatment; however, we observed higher serum ACE2 in females than in male WKY rats.

The effective reduction of ACE1 and ACE2 protein expression by HCQ in the lungs is observed in both sexes in SHRs and in female normotensive WKY rats. However, it is not observed in the male normotensive WKY, where both proteins are increased in the lung. ACE1 and ACE2 mRNA expression is differently regulated in male and female SHRs by HCQ in the lung tissue. In normotensive WKY rats, no differences in ACE1 and ACE2 mRNA expression were observed.

The reduction of ACE1 and ACE2 protein expression in males, and the discrete increase in female adipose tissue of SHRs, demonstrate sexual dimorphism in response to HCQ treatment in SHR. On the other hand, we observed increased ACE1 protein expression in both sexes, increased ACE2 protein expression in males, and a reduction in female normotensive WKY rats, which also show sexual dimorphism in response to HCQ in WKY rats. ACE1 and ACE2 mRNA expression were unchanged in the adipose tissue of the SHRs. At the same time, they increased significantly in males. They decreased in female WKY rats, demonstrating different gene regulation between strains, and there is sexual dimorphism.

Our study demonstrates that losartan effectively reduces ACE2 in the blood, lung, and adipose tissue; however, due to sexual dimorphism, losartan decreases ACE2 in males but not as much in female SHRs.

Although captopril inhibits ACE1 in lung tissue, it increases ACE2 in the male lung, decreases it in female SHRs, and decreases ACE2 in both sexes in SHR in the adipose tissue. Captopril promotes a decrease in ACE2 in tissues effectively in the female but not in male SHR, which this increase could be indicative of significant susceptibility to COVID‐19 infection. Hydroxychloroquine decreases ACE2 in the lung of both sexes and adipose tissue of males but female SHR. In the normotensive rats, Hydroxychloroquine effectively inhibits ACE2 in females and increases it in males WKY. Therefore, Hydroxychloroquine effectively decreases ACE2 in hypertensive male rats and normotensive female rats. On the other hand, it is essential to consider the increase in ACE2 in tissues of hypertensive female and normotensive male rats because it explains that the drug can increase susceptibility to COVID‐19 infection.

These results demonstrate the heterogeneity of HCQ action on ACE2 receptors related to tissues, gender, and healthy condition. In fact, previous studies suggest men are more susceptible to contracting SARS‐CoV‐2 and exhibit higher mortality rates compared to women (Gemmati et al., 2020). Our results also indicate that ACE2 protein expression not only varies for each tissue analyzed but also according to the male and female sex. We believe that the potential reasons for these differences cannot be attributed solely to the fact that the ACE2 gene is in the X‐chromosome (Gemmati et al., 2020), but it is likely that various factors are involved including sex hormones, innate immune system (Taslem Mourosi et al., 2022), baseline blood pressure levels, etc. that influence ACE1/ACE2 equilibrium in both sexes (Gemmati et al., 2020).

Previous studies from our laboratory and others show that the renin‐angiotensin system in rats functions in a similar manner when compared to humans. Therefore, we decided to investigate the impact of ACEi and ARBs in addition to hydroxychloroquine in ACE1 and ACE2 regulation in various tissues in hypertensive and normotensive rats. However, we do recognize that our results will need to be reproduced in humans.

## 5 CONCLUSION

The present results using losartan, captopril, and Hydroxychloroquine help explain the variable effects of these drugs on ACE2 regulation, which may influence the effectiveness of COVID‐19 treatments. Moreover, our study suggests that Hydroxychloroquine is helpful as a chemoprophylaxis treatment proposed by the Indian Council of Medical Research due to its impact on ACE2 receptors. However, we observed that ACE2 increased in male normotensive WKY rats with hydroxychloroquine treatment, indicating potential differential effects in hypertensive versus normotensive animals. Thus, these results further our understanding of the impact of anti‐hypertensive agents that inhibit the RAS and of Hydroxychloroquine in hypertension, suggest potential sex differences on ACE regulation in various tissues, which may help guide future clinical studies on COVID‐19 infection.

## AUTHOR CONTRIBUTIONS

Beatriz Santos Geoffroy Corrêa: investigation; Silvana de Barros: investigation; Julia Braga Vaz: investigation; Maria Angelica Peres: conceptualization; Mayara Klimuk Uchiyama: conceptualization; Alexandra Alves da Silva: conceptualization, reviewer, and editing; Luzia Naoko Shinohara Furukawa: conceptualization, methodology, formal analysis, investigation, data curation, writing original draft, reviewer and editing, supervision, project administration, and funding acquisition.

## FUNDING INFORMATION

This work was supported by a grant from the #HCcomVida, Fundação Faculdade de Medicina (grant number 1/2020).

## ETHICS STATEMENT

The experimental protocol was approved by the CEUA (Ethics Committee on the Use of Animals from the University of São Paulo School of Medicine no. 1535/2020). All procedures with animals followed the Ethics committee recommendations strictly.

## CONFLICT OF INTEREST STATEMENT

None.
